# Immune Responses Induced by pVAX/*Tg*ERK7 against *Toxoplasma gondii* Infection in BALB/c Mice

**Published:** 2019

**Authors:** Hai-Ting GUO, Zhong-Yuan LI, Jin-Lei WANG, Zhao-Yu GENG, Xing-Quan ZHU

**Affiliations:** 1. Guangxi Key Laboratory for Brain and Cognitive Neurosciences, Guilin Medical College, Guilin, China; 2. College of Biological Science and Technology, Heilongjiang Bayi Agricultural University, Daqing, China; 3. State Key Laboratory of Veterinary Etiological Biology, Key Laboratory of Veterinary Parasitology of Gansu Province, Lanzhou Veterinary Research Institute, Chinese Academy of Agricultural Sciences, Lanzhou, China; 4. College of Animal Science and Technology, Anhui Agricultural University, Hefei, China

**Keywords:** *Toxoplasma gondii*, toxoplasmosis, ERK7, DNA vaccine, Immune response

## Abstract

**Background:**

*Toxoplasma gondii* can infect all the warm-blooded vertebrates and cause serious toxoplasmosis. Extracellular signal-regulated kinase 7 in *T. gondii* (*Tg*ERK7) mediated the proliferation of this parasite may be a potential vaccine candidate. Thus, immune responses induced by *Tg*ERK7 were investigated in this study using a DNA vaccine strategy.

**Methods:**

pVAX/*Tg*ERK7 plasmid was constructed and used to immunize BALB/c mice for three times with two-week intervals. The challenge and the investigation of humoral and cellular immune responses were performed at two weeks post the last immunization, and the survival times of the infected mice were daily recorded until all of them were dead.

**Results:**

The innate immune response with higher concentrations of IFN-γ, TNF-α, IL2 and IL12p70 in sera (*P* < 0.05), and the adaptive immune responses were evoked by the DNA immunizations, including specific antibody, lymphocyte proliferation, and the CD3e+CD4+ and CD3e+CD8a+ T cell-mediated response effects. Interestingly, no significant difference was detected in their survival times among all the experimental groups of mice that were challenged with GT1 tachyzoites or PRU cysts (*P*>0.05).

**Conclusion:**

The successive immunizations with pVAX/*Tg*ERK7 can provoke the innate and adaptive immune responses of BALB/c mice, whereas the DNA vaccine-induced immunological efficacy is not sufficient for complete protection the host against *T. gondii* infection.

## Introduction

T*oxoplasma gondii* is an important protozoan parasite that can infect almost all the animals from avian species to mammals including approximately 30% human population (1). This parasite has received fundamental medical and scientific attentions as it can cause serious toxoplasmosis, especially for pregnant women and immunocompromised individuals (2). Immunization would be the ideal strategy to prevent the spreading of this parasite and many *Toxoplasma* antigens have been attempted as the vaccine candidates against *T. gondii* infection in mice (3).

Mitogen-activated protein (MAP) kinases expressed in all the eukaryotes constitute a family of proline-directed serine/threonine kinases, and play important roles in the regulation of cell proliferation, differentiation, apoptosis and stress responses through phosphorylation and dephosphorylation (4, 5). The homologues of MAP kinases have been discovered, and been further studied especially for the protozoan parasites. In *Leishmania mexicana,* two homologues of MAP kinases were identified, LmxPK4 involved in the differentiation and virulence of this parasite and LmxMPK9 associated with the flagellum bio-genesis and maintenance (6, 7). In *Giardia lamblia*, the homologues of extracellular signal-regulated kinase 1 (ERK1) and ERK2 were thought to mediate the transformation from trophozoites to cysts (8). Furthermore, Pfmap2 participates in the regulation of the asexual cycle of *Plasmodium falciparum* (9, 10).

Two homologues of MAP kinases were identified in *T. gondii* as yet (11, 12). *Tg*MAPK1, the homologue of p38α MAPK, plays a critical role in the development, growth and the conversion of *T. gondii* from tachyzoite to bradyzoite *in vitro* (13, 14). Another homologue *Tg*ERK7, alternate name *Tg*MAPK2, was confirmed as an important molecule that participates in the regulation of intracellular proliferation of *T. gondii*, suggesting that it might be a potential vaccine candidate for the prevention and control of this parasite (15). Therefore, the immune responses induced by *Tg*ERK7 were investigated using a DNA vaccine strategy in BALB/c mice, the verifiable animal model of *T. gondii* (16).

## Materials and Methods

### Ethics statement

All the experiments in this study were approved by the Animal Ethics and Administration Committee of Lanzhou Veterinary Research Institute, Chinese Academy of Agricultural Sciences (Approval No. LVRIAEC2012-011).

### Animals and parasites

BALB/c mice of 6–8 week old (20.0 ± 2.0 g) in specific-pathogen-free (SPF) grade were purchased from Lanzhou University Laboratory Animal Center (Lanzhou, China), and were subsequently bred without any treatment in the High-density Touch Screen Mouse IVC (FENGSHI, Suzhou, China) for one week to eliminate the stress reaction.

Tachyzoites of *T. gondii* GT1 strain (Geno-type I) were maintained by serial passages in African green monkey kidney (Vero) cells as previously described (17). The survival BALB/c mice challenged with 10 cysts of PRU strain (Genotype II) by oral one month before were executed by cervical spine dislocation for the serial passages. The obtained PRU cysts and GT1 tachyzoites were used for challenge of immunized BALB/c mice and preparation of *Toxoplasma* lysate antigen (TLA).

### Construction of DNA vaccine

Total RNA of GT1 tachyzoites was extracted using TRIzol reagent (Invitrogen, Carlsbad, USA). A pair of specific primers (forward primer: 5′-GGGGTACCATGAGTGACGAGGTC GACAAAC-3′; reverse primer: 5′-GCTCTAGATCAGCTGTTGTATGTCTTGGAC-3′) was designed to clone the coding sequence of *Tg*ERK7 gene (ToxoDB: TGGT1_233010), in which *KpnI* and *XbaI* restriction sites were marked with underlines, respectively.

RT-PCR amplification was performed as the following protocols: incubation at 50.0 °C for 30 min; inactivation at 95.0 °C for 2 min; denaturation at 95.0 °C for 10 min followed by 35 cycles composing of 95.0 °C for 45 s, 56.0 °C for 30 s and 72.0 °C for 2 min; final extension step at 72.0 °C for 10 min. The products were inserted into pMD18-T vector (TaKaRa), formed pMD/*Tg*ERK7. *Tg*ERK7 fragments were then cut from pMD/*Tg*ERK7 and subcloned into pVAX I vector (Invitrogen) using T4 DNA ligase (TaKaRa), generated pVAX/*Tg*ERK7. Sequence accuracy was confirmed by specific PCR, DNA sequencing and double digestion.

### Antigenic fragment prediction and antibody preparation

A short antigenic peptide (N′-3-DEVDKHVLRKYD-14-C′) from the N-terminal α-helical region of *Tg*ERK7 protein was elaborately selected as previously described (18, 19). The peptide synthesis and conjugation with Keyhole Limpet Hemocyanin (KLH) were done by SBS Genetech Co., Ltd. (Beijing, China), which was subsequently used for antibody preparation (α*Tg*ERK7) according to the standard 70-day rabbit immunization protocol of Thermo Fisher Scientific Inc. (https://corporate.thermofisher.com/).

### Detection of TgERK7 protein expressed in HEK293 cells

The indirect immunofluorescence was performed in the human embryonic kidney cells (HEK293) to determine whether *Tg*ERK7 protein could be expressed in the eukaryotic cells (20). Briefly, the cells grown in twelve-well plate were transfected with 5 μg of pVAX/*Tg*ERK7 plasmid using lipofectamine 2000 (Invitrogen). 72 h post transfection, the samples were incubated with α*Tg*ERK7 and secondary antibody for 1 h, respectively. The antibody dilutions were as follows: α*Tg*ERK7, 1:250; donkey anti-rabbit Alexa 488 (Invitrogen), 1:1000. The cells transfected with empty pVAX I vector were used as the negative control. Results were recorded using a fluorescence microscope (Olymplus IX 51) and a 2.8 megapixel color CCD (pixel resolution max. 1920 × 1440).

### Immunization and challenge

All the BALB/c mice were randomly separated into four groups (26 mice each group, Table 1), and the procedures of immunization and challenge performed in mice were similar to the precious description (21). The blood samples were randomly collected from the tail vein of three mice each group at 0, 2, 4 and 6 weeks for the antibody and cytokine detection. Notably, the samples from only three mice each group before immunization were used as negative controls. Moreover, three mice each group were humanely handled to death at 14 day post the last immunization, and their spleens were aseptically removed to prepare the single-cell suspensions for T lymphocyte subclass and lymphocyte proliferation analyses.

**Table 1:** Summary of treatments for immunization and challenge performed in BALB/c mice groups

***Group***	***Treatments (of 100 μl PBS)***	***Total sample size***	***Route of administration***	***Mice number in LAC ^a^***	***Mice number in CIR ^b^***	***Mice number in challenges***
I	100 μg pVAX/*Tg*ERK7	26	Thigh muscle	3	3	10 ^c^ , 10 ^d^
II	100 μg pVAX I	26	Thigh muscle	3	3	10 ^c^, 10 ^d^
III	100 μl PBS	26	Thigh muscle	3	3	10 ^c^, 10 ^d^
IV	Control	26	-	3	3	10 ^c^, 10 ^d^

^a^ To assess the levels of antibodies and cytokines (LAC) in sera, blood samples were randomly collected from three mice each group prior to the challenge and every immunization.

^b^ To assess the cellular immune responses (CIR), spleens from three mice each group were aseptically removed at 6 weeks post the first immunization.

^c^ Two weeks after the last immunization, ten mice each group were challenged intraperitoneally with 1×10^3^ tachyzoites of *T. gondii* GT1 strain.

^d^ Two weeks after the last immunization, another ten mice each group were challenged intragastrically with 20 cysts of *T. gondii* PRU isolate.

Ten mice in all the groups were intraperitoneally challenged with 1 × 10^3^ tachyzoites of *T. gondii* GT1 strain, and another ten mice were inoculated with 20 cysts of PRU isolate by oral at two weeks post the last immunization (Table 1), and their survival times were recorded daily until all the mice were dead.

### Antibody and cytokine assays

The short *Tg*ERK7 peptide, ELISA plate and SBA Clonotyping System-HRP Kit (Southern Biotech, Birmingham, USA) were used for detection of the specific anti-*Tg*ERK7 and total IgG antibodies. Briefly, ELISA plate was coated with 1.0 μg of capture antibody provided by the kit or equal short peptide diluted into the sterile PBS (pH 7.4) via incubation overnight at 4 °C. The following protocols including the antibody incubation were performed as the previous description (22). The absorbance was recorded at 450 nm at 10 min and 20 min after substrate addition using a ELISA reader (Bio-TekEL × 800, USA).

For cytokine, the levels of tumor necrosis factor-alpha (TNF-α), monocyte chemoattractant protein-1 (MCP-1), interferon-gamma (IFN-γ), interleukin 2 (IL2), IL4, IL6, IL10, IL12p70 and IL23 in sera separated at 6 weeks post the first immunization were detected by the LEGEND MAX^TM^ Mouse ELISA kits (Biolegend, San Diego, USA). Their concentrations were calculated according to the standard curves that were drawn with the standard samples provided by the kits. The data was recorded by the reader (Bio-TekEL × 800, USA) at 450 nm within 30 min after stop buffer addition.

### Lymphocyte proliferation and flow cytometry analyses

Spleens of three mice each group were aseptically removed and the single-cell suspensions were prepared for lymphocyte proliferation assay as previously described (23). Briefly, the cells were re-suspended in RPMI 1640 medium (Hyclone, Logan, USA) supplemented with 10% fetal bovine serum (FBS) (Invitrogen), followed by counted with 0.04% trypan blue (Bio-Rad, California, USA), plated in 96-well costar plates (5 × 10^5^ cells/well) and incubated with TLA (10 μg/ml), concanavalin A (ConA, 5 μg/ml, Sigma, Missouri, USA) or medium alone at 37 ^o^C in an atmosphere of 5% CO_2_/air. Lymphocyte proliferation evaluated by stimulation index (SI) was determined by 3-(4,5-dimethylthylthiazol-2-yl)-2,5-diphenyltetrazolium bromide (MTT, 5 mg/ml, Sigma). SI was calculated as the ratio of OD_570_ value of wells containing stimulus (TLA or ConA) compared with the medium (OD_570M_).

For flow cytometry assay, 1 × 10^5^ cells were incubated with PE anti-mouse CD3e, APC anti-mouse CD4 and/or FITC anti-mouse CD8a (eBioscience, San Diego, USA) at 4 °C for 30 min in dark followed by washed with 2 ml of PBS (pH 7.4), fixed by FACScan buffer and blocked with 2% paraformaldehyde (17). Data was collected using a FACScan flow cytometer (BD Bio-sciences, USA) and the SYSTEM II software (Coulter).

### Statistical analysis

All the collected data from three independent replicates for each treatment was analyzed using the SPSS13.0 Data Editor (SPSS Inc., Illinois, USA). The statistical difference between any two groups was evaluated by the student's t-tests if their variances were equal measured by *F*-test. The results displayed with the mean with standard error (Mean ± S.E.) were considered to be statistically different if ^*^P<0.05, or significantly different if ^**^P<0.01.

## Results

### Expression of TgERK7 recombinant protein in eukaryotes

**Fig. 1:**
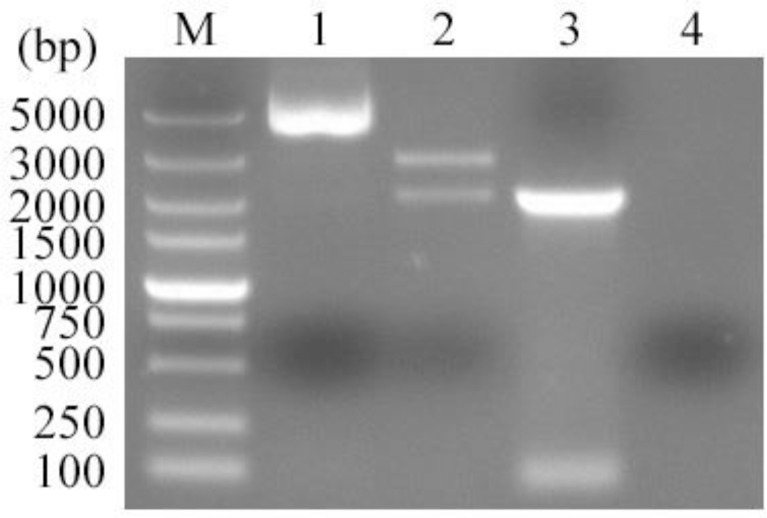
Identification of pVAX/*Tg*ERK7 with *Kpn*I/*Xba*I restriction enzymes. Two bands (~2999 bp and ~2079 bp, respectively) were detected in the enzyme-digested product, suggesting that pVAX/*Tg*ERK7 was successfully constructed. Lanes: M, DL5000 DNA marker; 1, pVAX/*Tg*ERK7; 2, Digestion product of pVAX/*Tg*ERK7 with *Kpn*I/*Xba*I; 3, PCR product of *Tg*ERK7 gene; 4, Negative control

**Fig. 2:**
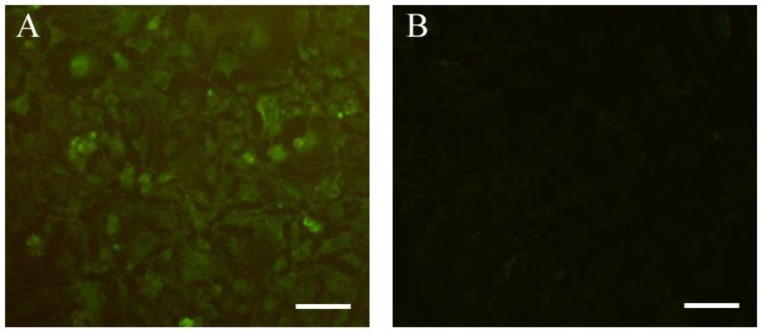
Expression of *Tg*ERK7 recombinant protein *in vitro*. Some substance with specific green fluorescence can be detected in the pVAX/*Tg*ERK7-transfected HEK293 cells (A); no light was in the cells with empty pVAX I (B). White bar = 50 μm

To determine whether the *Tg*ERK7 protein was expressed by pVAX/*Tg*ERK7 in eukaryotic cells or not, which was identified using *Kpn*I and *Xba*I double digestions (Fig. 1), the immunofluorescence assay were performed in HEK293 cells. Our results showed that the specific green fluorescence can be only detected in the pVAX/*Tg*ERK7-transfected cells (Fig. 2. A), whereas no light was in control (Fig. 2. B), suggesting that *Tg*ERK7 recombinant protein can be expressed in the eukaryotic cells.

### Levels of antibodies and cytokines induced by pVAX/TgERK7

Specific anti-*Tg*ERK7 and total IgG antibodies in sera were examined to assess the humoral immune response induced by DNA immunization.

**Fig. 3:**
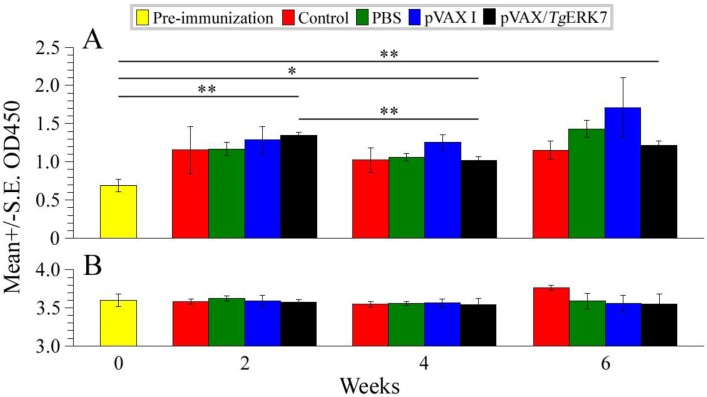
Levels of antibodies in serum samples induced by DNA immunization. The specific anti-*Tg*ERK7 (A) and total IgG (B) antibodies in sera of mice were examined. The data indicated that the specific antibody titers of the immunized mice did not incrementally increase compared with the groups of pVAX I, PBS and blank control; no statistical difference of total IgG antibodies were among all the groups of mice (*P* > 0.05). ^*^P < 0.05, ^**^P < 0.01

We found that the specific antibody titers of pVAX/*Tg*ERK7-immunized mice did not incrementally increase in comparison with their controls (pVAX I, PBS, and blank control), though the levels of the specific anti-*Tg*ERK7 IgG in the experimental group were higher compared with that of the pre-immunization mice (Fig. 3. A). Meanwhile, no statistically different total IgG antibodies were observed between any two groups of the immunized and non-immunized mice (*P* > 0.05) (Fig. 3. B), suggesting that the slight humoral immune responses of mice were induced by the DNA immunizations.

**Fig. 4:**
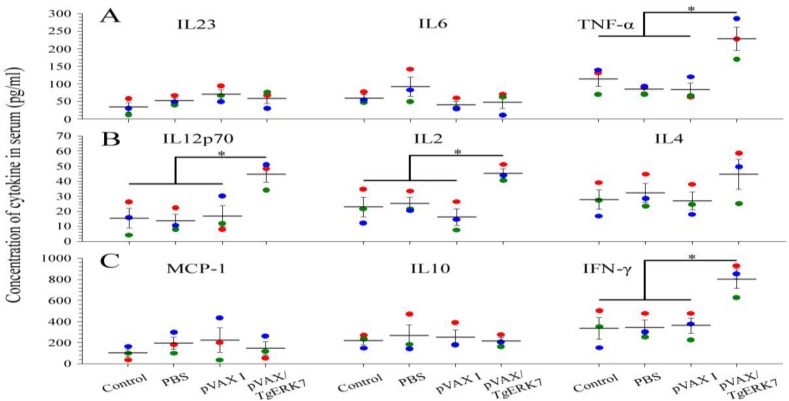
Concentrations of cytokines in sera that were separated at two weeks post the last immunization. During the nine kinds of cytokines (A: IL23, IL6 and TNF-α; B: IL12p70, IL2 and IL4; C: MCP-1, IL10 and IFN-γ, respectively), only four kinds of them increased significantly in comparison with their control groups (P < 0.05)

The serum samples were also used to measure the concentrations of cytokines, the data of which indicated that the levels of IFN-γ, IL2, IL12p70 and TNF-α in sera of the immunized mice significantly increased by contrast with their three control groups (*P* < 0.05), whereas not any statistical difference was detected in the MCP-1, IL4, IL6, IL10 and IL23 among all the groups (Fig. 4).

### Cellular immune responses

**Fig. 5:**
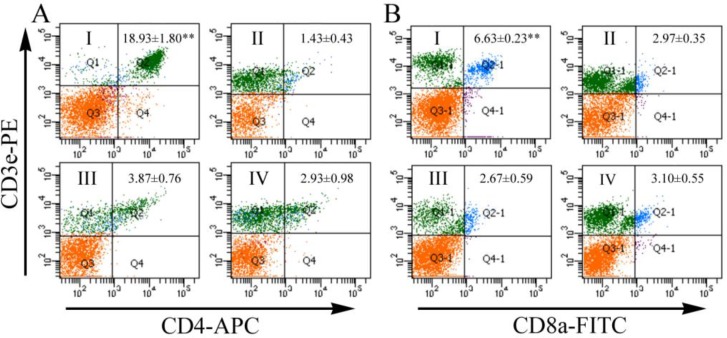
Detection of lymphocyte subsets in spleen using flow cytometry. The number of groups were marked in the regions of Q1 or Q1-1, and their percentages of CD3e+CD4+ T (A) and CD3e+CD8a+ T (B) lymphocytes presented with the mean ± S.E. were marked in Q2 or Q2-1, respectively. ^**^ indicates P values less than 0.01

**Fig. 6:**
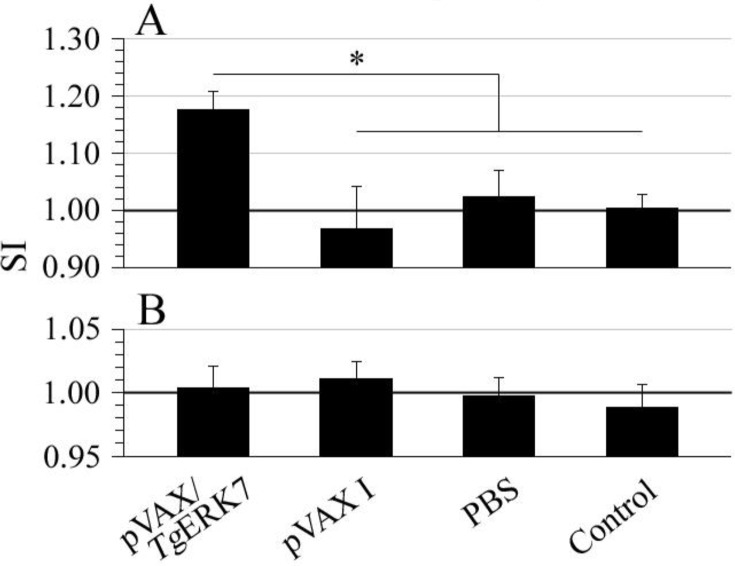
Proliferation analysis of the lymphocytes in spleen tissues using MTT method. The single-cell suspensions prepared at 14 days post the last immunization were stimulated with TLA (A) or ConA (B), and their stimulation index (SI) was analyzed in comparison with the three controls. ^*^P < 0.05

The cellular immune responses induced by pVAX/*Tg*ERK7 were evaluated by the contents of CD3e+CD4+ T and CD3e+CD8a+ T lymphocytes in spleens, and the results showed that both CD3e+CD4+ T and CD3+CD8a+ T cells of the immunized mice increased significantly in comparison with the controls (*P* < 0.01), and no difference was between any two groups of the controls (Fig. 5). The proliferation of lymphocytes in spleen tissues separated at 14 days post the last immunization was also performed in the study. For TLA stimulus, SI value of the mice immunized with pVAX/*Tg*ERK7 (1.18 ± 0.03) was statistically higher than the groups of pVAX I (0.97 ± 0.07), PBS (1.03 ± 0.04) and blank control (1.00 ± 0.03) (*P* < 0.05); but for ConA, no difference was among all the experimental groups (Fig. 6).

### Protective efficacy of pVAX/TgERK7-immunized mice against T. gondii

**Fig. 7:**
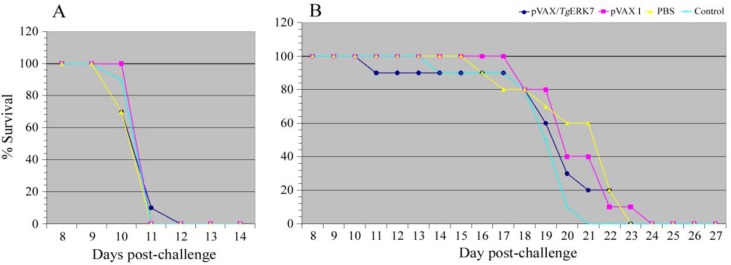
Protective efficacy induced by pVAX/*Tg*ERK7 immunizations against *Toxoplasma* infections in the mice. (A) The survival curves of mice challenged with 1 × 10^3^ tachyzoites of *T. gondii* GT1 strain. All the three control groups of mice (pVAX I, PBS and blank control) died within 11 days post challenge. (B) The survival curves of mice challenged with 20 cysts of *T. gondii* PRU isolate. All the cyst-infected mice including the pVAX/*Tg*ERK7-immunized group died within 24 days post challenge. Not any statistical difference was detected among all the groups (P > 0.05)

All the immunized and non-immunized mice were finally challenged with the virulent tachyzoites of GT1 strain (Genotype I) and cysts of PRU isolate (Genotype II) to investigate the immunological efficacy of the mice against the *T. gondii* infections induced by the successive DNA immunizations. The survival times of all the groups were recorded daily until the mice were all dead. Our data showed that no significantly different survival times were detected in the pVAX/*Tg*ERK7-immunized mice (10.8 ± 0.2 d for GT1, and 19.4 ± 1.1 d for PRU) by contrast with their controls (pVAX I: 11.0 ± 0.0 d for GT1, and 20.6 ± 0.6 d for PRU; PBS: 10.7 ± 0.2 d for GT1, and 20.6 ± 0.8 d for PRU; blank control: 10.9 ± 0.1 d for GT1, and 19.0 ± 0.6 d for PRU, respectively). All the mice of controls died within 11 days (10/10) for GT1 strain, and 24 days (10/10) for PRU isolate after challenge, respectively (Fig. 7).

## Discussion

The protozoan parasite *T. gondii* can infect almost all the warm-blooded vertebrates including animals, birds and humans, causing serious toxoplasmosis (24, 25). Many attempts have been made to restrain the development and spreading of this parasite. In the last few years, the effective subunit or live-attenuated *T. gondii* strain vaccines against *T. gondii* was identified, but they are not applied invariably because of their expensiveness, side effects and short shelf life (26). Thus the current efforts have been devoted to the exploration of DNA vaccines (3, 27). DNA-delivered vaccines based on pVAX I ability to express many genes in eukaryotic cells, can evoke long-time humoral and cellular immune responses (21, 28). Thus, this efficient eukaryotic system was employed in this study to express the *Tg*ERK7 protein, and its antigenicity was demonstrated to be satisfactory with successful expression of *Tg*ERK7 recombinant protein in eukaryotes using the indirect immunofluorescence assay.

As previously described, during natural invasion and artificial inoculation of *T. gondii*, both the innate and adaptive immune responses in host determined the outcome of *T. gondii* infection (29). Meanwhile, this parasite was able to invading to host cell, Th1-type response characterized by the production of early IL12 and subsequent IFN-γ (30). In order to determine the levels of innate immune response induced by the successive pVAX/*Tg*ERK7 immunizations, besides IL12p70 and IFN-γ, another seven kinds of cytokines associated with innate immune responses such as TNF-α (31), MCP-1 (32), IL2, IL4, IL6, IL10 and IL23 before challenge were measured (29, 33, 34). Our data showed that the levels of four kinds of cytokines (IFN-γ, TNF-α, IL2 and IL12p70) in the pVAX/*Tg*ERK7-immunized group statistically increased in comparison with the three controls, whereas no difference was detected among all the groups for IL4, IL6, IL10, IL23 and MCP-1. Although some kind of the depressed cytokine might be beneficial for generating the safe and effective therapeutic vaccines under the viral infection and cancer (35), but these results suggested that the innate immune response was not significantly evoked by DNA immunization and the concentration of down regulators including IL4 and IL10 did not increase in contrast to that of the controls (36).

The critical function of specific antibody in serum has been recognized as one of the most important indexes to evaluate the levels of adaptive immune responses to *T. gondii* infection for a long time (37, 38). In comparison with a previous study (21), the level of specific antibody in sera was elevated slightly by the serial stimulation with pVAX/*Tg*ERK7 compared with the pre-immunization, and no difference was observed in total IgG antibody among all the groups, suggesting that the specific anti-*Toxoplasma* antibodies can be elicited by the DNA immunizations, whereas their levels were mainly dependent on the categories of the *T. gondii* antigens.

In order to further explore the adaptive immune responses induced by pVAX/*Tg*ERK7 immunization, several parameters involved in cellular immune responses were also measured in this study (29, 39). In agreement with our previous studies of MIC8 and eIF4A (17, 40), the T cell responses could be also significantly elicited by immunization with *Tg*ERK7, showing the increased percentages of CD3e+CD4+ T and CD3e+CD8a+ T cell subsets in spleen tissues. Interestingly, a slight lymphocyte proliferation was only detected in the wells containing specific TLA stimulus but not with ConA. These results suggested that the specific activation of CD3e+CD4+ T and CD3e+CD8a+ T cells might be in synergy to the further contribution of cytotoxic activity against *T. gondii* infections.

Finally, BALB/c mice susceptible to this protozoan parasite were employed to determine whether the levels of innate and adaptive immune responses induced by pVAX/*Tg*ERK7 immunizations were beneficial for the protection against *T. gondii* infections (16). We found that there was no significant difference in survival times among all the groups of mice challenged with GT1 strain (Genotype I) or PRU isolate (Genotype II), which were distinguishable from the previously described (17, 22).

## Conclusion

Although the inflammatory factors, specific antibody, lymphocyte proliferation, and the CD3e+CD4+ and CD3e+CD8a+ T cell-mediated response effects of BALB/c mice were statistically induced by pVAX/*Tg*ERK7 immunization, but there was no difference for the survival times against *Toxoplasma* infections between the experimental group and its control groups. Taken together, we have demonstrated that the successive immunizations with pVAX/*Tg*ERK7 can provoke the innate and adaptive immune responses of mice, whereas these immune responses are not sufficient to protect the host against the infections of *T. gondii*.
